# Plate fixation of the symphysis after symphysiotomy: A case report

**DOI:** 10.1016/j.tcr.2026.101387

**Published:** 2026-05-21

**Authors:** B.W. Hepkema, L.R. Werf van der, E. Nol, W. Wegdam, D. Embden van

**Affiliations:** a AmsterdamUMC location AMC, Department of Physical Medicine and Rehabilitation, Meibergdreef 9, Amsterdam, the Netherlands; b AmsterdamUMC location AMC, Department of Surgery, Section Trauma Surgery, Meibergdreef 9, Amsterdam, the Netherlands; c Zaans Medisch Centrum, Department of Gynaecologie, Julianaplein 58, Zaandam, the Netherlands

**Keywords:** Symphysiotomy, Symphysiolysis, Post-partum pelvic instability, Surgical fixation, Pelvic surgery

## Abstract

**Background:**

Symphysiotomy is a rarely performed obstetric procedure with known risks of long-term maternal morbidity.

**Case:**

We present the case of a 26-year-old woman who developed pelvic instability following a symphysiotomy performed during a delivery complicated by shoulder dystocia and neonatal death. Diagnostic imaging revealed pathological mobility of the pubic symphysis. Surgical stabilization with plate osteosynthesis via a modified Stoppa approach resulted in early recovery with almost normal functional outcomes in the first weeks after surgery.

**Conclusion:**

Persistent pelvic instability following symphysiotomy can significantly impair function and quality of life. This case highlights that surgical fixation of the pubic symphysis, can lead to full functional recovery. Early recognition of patients who are likely to benefit from surgery is important. Early surgical intervention may be essential for quick recovery and restore mobility, especially in cases of marked diastasis.

## Introduction

Symphysiotomy is a rarely performed obstetric intervention in which the pubic symphysis is surgically separated to increase the pelvic diameter and facilitate vaginal delivery in cases of obstructed labor such as shoulder dystocia. Historically, the procedure served as an alternative to cesarean delivery in contexts where access to surgical care was limited and surgical risks such as infection, anesthetic complications, or hemorrhage.

Although in practice symphysiotomy has been largely abandoned in high-income countries since the mid-20th century, it continues to be performed in certain low-resource settings as a life-saving intervention [1], [2].

Symphysiotomy carries a significant risk of maternal morbidity. Long-term sequelae described in the literature include varying degrees of pelvic girdle dysfunction, including instability, pain during mobilisation, altered gait, and urinary tract symptoms such as incontinence [3]. Some retrospective studies suggest that physical function and quality of life may be severely impaired in a subset of women following the procedure [4].

Most cases of post-symphysiotomy pelvic pain and instability are managed conservatively through physiotherapy and support devices. However, surgical stabilization may be required in severe cases. The literature on surgical management following symphysiotomy remains sparse, and clinical guidance is largely based on case reports and expert opinion [5]. Additionally, current national obstetric guidelines, such as those from the Dutch Society of Obstetrics and Gynaecology (NVOG), do not include specific protocols regarding treatment after symphysiotomy.

In this report, we describe the case of surgical treatment of a woman presenting with pelvic instability and functional impairment following symphysiotomy. We discuss the clinical course, imaging findings, and the rationale for surgical stabilization. Especially, this case highlights the functional outcomes after surgical intervention.

## Case presentation

A 26-year-old primigravida had an uncomplicated pregnancy without risk factors for shoulder dystocia such as fetal macrosomia. She delivered at a community hospital, where a shoulder dystocia occurred. Standard maneuvers, including suprapubic pressure and the McRoberts maneuver, were unsuccessful. Second-line maneuvers (delivery of the posterior arm and internal rotational maneuvers in both supine and all-fours position) also failed. Within five minutes of onset, a symphysiotomy was performed, after which the infant was delivered.

The mother was transferred to the operating room for closure of the symphysiotomy incision and assessment of urethral integrity. A pelvic stabilization device (T-Pod Responder) was applied. Her medical history was otherwise unremarkable, and she was physically active, participating in sports and gym training.

One week postpartum, because of pelvic pain and functional instability she was referred to the pelvic and acetabular trauma team of an Academic Level 1 trauma center and high-volume referral center for pelvic and acetabular injuries.

At presentation in the referral hospital the patient reported symphyseal pain without radiation to the lower back or buttocks. She reported being able to walk only with small steps and using a pelvic support band. There was no fever or systemic illness. She had a recent episode of urinary tract infection (UTI) treated with antibiotics and reported ongoing hematuria.

On physical examination, she was able to walk a couple of steps with internal rotation of the knees, though with discomfort. The surgical wound appeared clean and non-inflamed. Cautious testing of the pubic symphysis provoked pain.

**Fig. 1:**
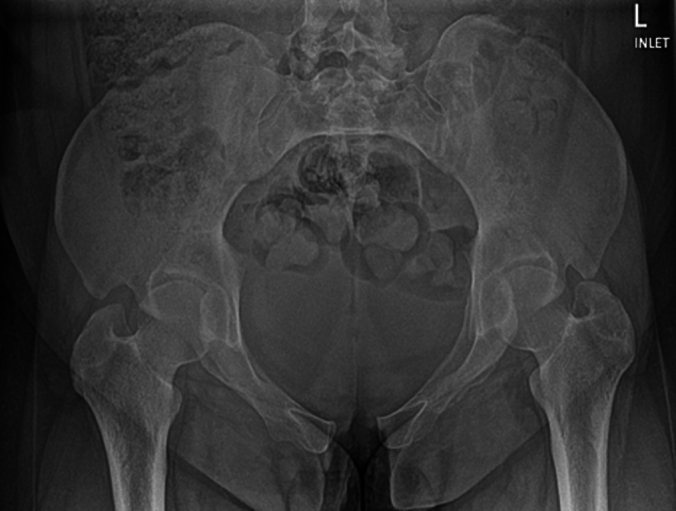
x-ray; an AP inlet view of the pelvic.

**Fig. 2:**
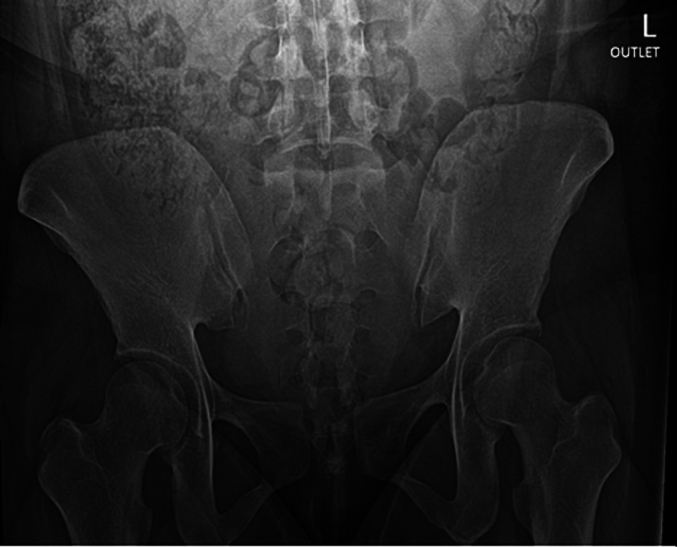
x-ray; an AP outlet view of the pelvic.

Pelvic imaging, including an inlet and outlet view on X-ray, showed a pubic symphysis diastasis measuring approximately 27 mm (Fig. 1, Fig. 2). The separation appeared disproportionately wide, raising concern for mechanical insufficiency. The CT scan showed a widening of 15 mm and no sign of sacroiliac joint widening or injury (Video 1).

**Fig. 3:**
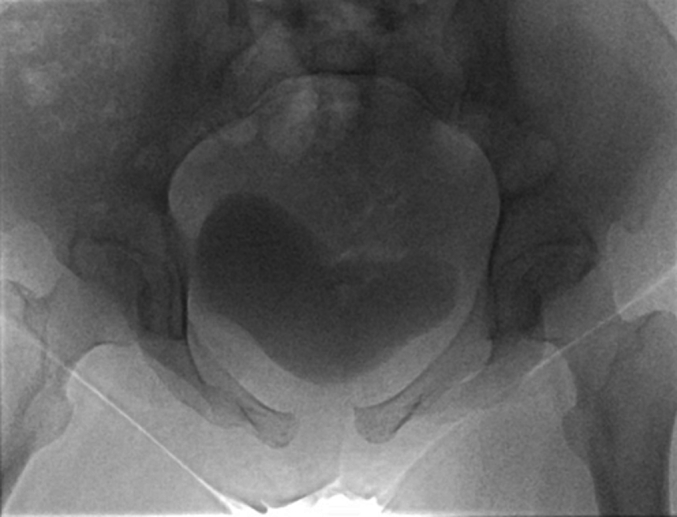
Fluoroscopic stress radiograph.

Because of the discrepancy between clinical presentation and imaging, a dynamic fluoroscopic stress test was performed. Fluoroscopic examination revealed pathological mobility of the pubic symphysis and a widened pubic symphysis during stress testing, confirming pelvic ring instability (Fig. 3, Video 2).

The urologist was consulted because of her post-partum urinary tract infection and the described high rates of urological injuries post- symphysiotomy. No injuries were demonstrated.

After weighting the various considerations in a multidisciplinary team and a shared decisions conversation with the patient, the patient decided to opt for surgical stabilization via an anterior intrapelvic (modified Stoppa) approach. A six-hole plate was placed across the symphysis with good bony purchase and mechanical grip. To reduce the risk of postoperative infection, a gentamicin-containing collagen sponge (Garacol) was placed on the plate beneath the fascia. Postoperatively, the patient was allowed with weight-bearing as tolerated, guided by pain and functional capacity. Kankanalu et al. (2021) [6] and Poole et al. (2022) [7] demonstrated that early unrestricted weight-bearing after stable fixation of the pelvic can be performed safely without increased loss of reduction or reoperation rates.

The day after operation the patient was able to walk with a walker and climb stairs.

At six-week follow-up, the patient reported only mild residual discomfort. She was able to walk for approximately one hour and demonstrated good single-leg stance on both sides. The six-week postoperative X-ray; showed a well-positioned plate over the symphysis pubis without signs of loosening or other hardware-related complications. The overall alignment of the anterior pelvic ring was preserved, and there were no signs of infection or implant failure.

**Fig. 4:**
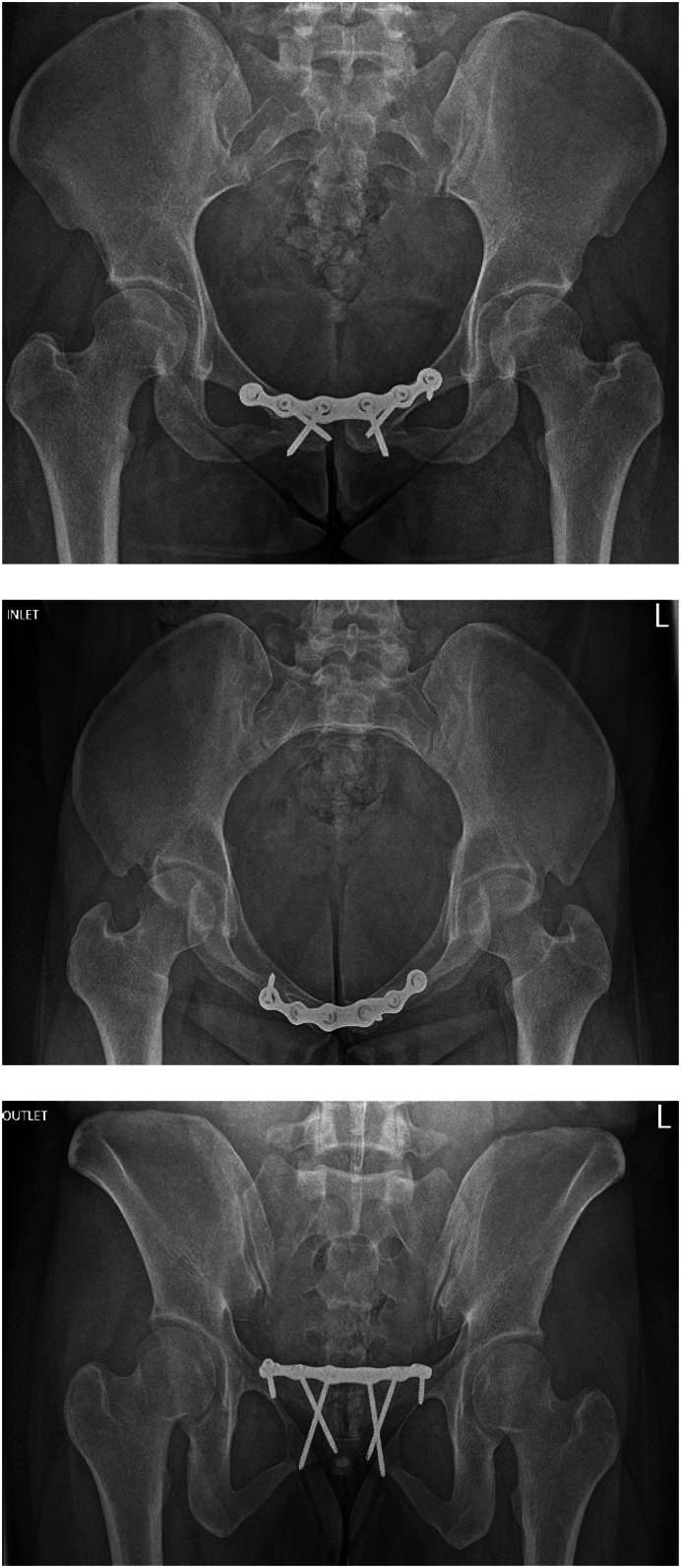
x-ray: an AP (1), inlet (2) and outlet (3) view of the pelvic.

At three months after surgery, the patient had returned to sports activities, including running and jumping, without any complaints. Functional outcome was assessed using a Dutch translated version of the Majeed Pelvic Score. The patient achieved a score of 100 out of 100 points, corresponding to an excellent functional outcome. Follow-up radiographs showed no signs of implant failure, loss of reduction, or other complications. Final AP, inlet, and outlet radiographs are presented in Fig. 4.

## Discussion

Symphysiotomy remains a controversial yet contextually relevant obstetric procedure, primarily used in cases of obstructed labor in low-resource settings. While it can be life-saving and offers advantages such as avoiding cesarean section and preserving the option of future vaginal delivery, long-term functional outcomes are variable and often underreported.

In this case, a 26-year-old woman was referred due to signs of severe pelvic instability one week after symphysiotomy. She underwent successful surgical stabilization via the Stoppa approach with plate osteosynthesis, leading to significant improvement in symptoms and function.

This is consistent with a case described by Chalidis et al. (2007), in which a primiparous woman developed a 4 cm symphyseal diastasis after emergency symphysiotomy for shoulder dystocia. Conservative treatment failed due to persistent pain and impaired mobility [8]. Surgical reconstruction via a Pfannenstiel approach with anatomical reduction, bone grafting, and dual plating resulted in full recovery without residual complaints. The authors emphasized the importance of early surgical intervention in cases of significant instability to ensure optimal functional outcomes and avoid long-term morbidity.

Another technically different but conceptually comparable case was presented by Sosnoski et al. (2023), involving a 44-year-old woman with postpartum pelvic instability. She underwent anterior plating of the pubic symphysis combined with bilateral sacroiliac screw fixation. In contrast to our case, which involved anterior fixation via the Stoppa approach alone, this approach addressed both anterior and posterior pelvic instability [9]. Both cases demonstrated favorable short-term functional outcomes, reinforcing the value of surgical stabilization—whether limited to the anterior pelvis or involving the entire pelvic ring—when conservative measures fail.

Support for surgical management also emerges from broader literature. In this context, Broekman et al. (1994) described a 38-year-old multiparous woman who underwent symphysiotomy following severe shoulder dystocia. Although the procedure enabled safe delivery of a 5020 g infant, the mother developed a vesicovaginal fistula that required surgical repair, and the neonate experienced a transient brachial plexus injury [10]. This case illustrates that while symphysiotomy may be necessary in emergency situations, it is not without significant maternal and neonatal risks.

In contrast to the positive outcomes of surgical stabilization, less favorable results were reported by Ersdal et al. (2008) in a cohort study of 34 women in Zimbabwe who had undergone symphysiotomy without surgical stabilization. Although the procedure successfully facilitated vaginal delivery, a considerable proportion of women reported ongoing pain and functional limitations during follow-up. Specifically, 23.5% experienced pain while walking and 29.4% reported dyspareunia, often due to pubic pain during abduction [11]. These findings underscore that spontaneous healing does not guarantee functional recovery and that surgical intervention may be warranted in selected cases to prevent chronic morbidity.

This is further supported by Björklund (2003), who reviewed the use of symphysiotomy throughout the twentieth century, particularly in Ireland and developing countries [12]. The author highlights that while symphysiotomy can be performed safely with minimal equipment and preserve future vaginal birth potential, poor surgical technique, inadequate training, and lack of follow-up care can lead to chronic pelvic pain, gait disturbances, and sexual dysfunction. Björklund stresses that long-term morbidity is likely underreported and deserves more clinical attention.

Taken together, these case reports, cohort data, and historical reviews suggest that surgical stabilization of the pubic symphysis should be considered in patients with persistent pelvic instability after symphysiotomy. Particularly when functional limitations are present, assessment of pelvic ring stability through fluoroscopic examination is recommended. In cases of pathological mobility of the pubic symphysis, early surgical intervention may help prevent long-term disability and restore mobility and quality of life.

## Conclusion

In line with the scarce existing literature, our findings support the recommendation that in cases of a widened pubic symphysis and/or a positive fluoroscopic examination, early surgical stabilization could be considered. Timely recognition of instability, combined with multidisciplinary follow-up and appropriate surgical intervention, may obtain early recovery, prevent long-term morbidity and restore quality of life after symphysiotomy.

## CRediT authorship contribution statement

**B.W. Hepkema:** Writing – review & editing, Writing – original draft. **L.R. Werf van der:** Writing – review & editing, Writing – original draft. **E. Nol:** Writing – review & editing. **W. Wegdam:** Writing – review & editing. **D. Embden van:** Writing – review & editing, Writing – original draft, Supervision, Conceptualization.

## Consent

Written informed consent was obtained from the patient for publication of this case report and accompanying images.

## Funding sources

This research did not receive any specific grant from funding agencies in the public, commercial, or not-for-profit sectors.

## Declaration of competing interest

All authors have nothing to declare.
